# Glucose is a key driver for GLUT1-mediated nanoparticles internalization in breast cancer cells

**DOI:** 10.1038/srep21629

**Published:** 2016-02-22

**Authors:** Leonardo Venturelli, Silvia Nappini, Michela Bulfoni, Giuseppe Gianfranceschi, Simone Dal Zilio, Giovanna Coceano, Fabio Del Ben, Matteo Turetta, Giacinto Scoles, Lisa Vaccari, Daniela Cesselli, Dan Cojoc

**Affiliations:** 1 PhD School of Nanotechnology, Department of Physics, Via Valerio 2, I-34127, University of Trieste, Trieste, Italy; 2 CNR–Institute of Materials, Area Science Park-Basovizza, S.S. 14, Km 163.5, I-34149 Trieste, Italy; 3 Department of Medical and Biological Sciences, Piazzale Kolbe 2, I-33100, University of Udine, Udine, Italy; 4 Elettra Synchrotron Trieste, SISSI beamline, Area Science Park-Basovizza, S.S. 14, Km 163.5, I-34149 Trieste, Italy

## Abstract

The mesenchymal state in cancer is usually associated with poor prognosis due to the metastatic predisposition and the hyper-activated metabolism. Exploiting cell glucose metabolism we propose a new method to detect mesenchymal-like cancer cells. We demonstrate that the uptake of glucose-coated magnetic nanoparticles (MNPs) by mesenchymal-like cells remains constant when the glucose in the medium is increased from low (5.5 mM) to high (25 mM) concentration, while the MNPs uptake by epithelial-like cells is significantly reduced. These findings reveal that the glucose-shell of MNPs plays a major role in recognition of cells with high-metabolic activity. By selectively blocking the glucose transporter 1 channels we showed its involvement in the internalization process of glucose-coated MNPs. Our results suggest that glucose-coated MNPs can be used for metabolic-based assays aimed at detecting cancer cells and that can be used to selectively target cancer cells taking advantage, for instance, of the magnetic-thermotherapy.

Around 1930, Otto Heinrich Warburg discovered that, even in the presence of oxygen, tumor cells undergo aerobic glycolysis rather than a normal oxidative phosphorylation^1^. Aerobic glycolysis produces just 2 molecules of ATP per molecule of glucose, while up to 36 ATP molecules are produced by oxidative phosphorylation, thus cancer metabolism and oncogenes have been investigated to better understand the reason why tumor cells, that require high ATP levels to supply their energy needs, take this pathway^2^. Nowadays it is clear that both normal and tumor cells are capable to switch oxidative pathway to overcome their energetic drawbacks, the former process by a finely regulated way whereas the second is allowed by a deregulated gene expression^3^,^4^. Although it is not clear whether the Warburg effect is the cause or the consequence of the genetic dysregulation^5^, the increased glucose metabolism of cancer cells has been used for diagnostics purposes, such as for the Positron Emission Tomography with the [^18^F]-Fluorodeoxyglucose ([^18^F]FDG)^6^,^7^. In a recent paper, Alvarez and co-workers demonstrated a high [^18^F]FDG uptake, by glucose specific transporter 1 (GLUT1), in aggressive Her2-positive mammary tumors^8^. Moreover, in this high grade cancer, it has been demonstrated that the aerobic glycolytic metabolism correlates with tumor aggressiveness^9^. GLUT1 protein is member of a family of glucose transporter molecules belonging to solute carrier 2A (SLC2A)^10^ and it is over-expressed in cell lines derived from highly aggressive tumors, both as mRNA^11^ and protein^12^. These and other works^13^,^14^ outlined the particular metabolic process characterizing the high aggressive cancer cells. Specifically targeting these cells by exploiting their metabolic pathways^15^,^16^, rather than using membrane receptors, represents one of the most interesting and promising approaches in cancer research, that could, for instance, help to overcome drug resistance^12^,^17^.

In this work we proposed a metabolic-based method to detect breast cancer cells with a basal phenotype (basal cells with mesenchymal features)^18^ and discriminate them, in a co-culture environment, from those with a luminal phenotype. MCF7 and MDA-MB-231 have been chosen as breast cancer cell lines representative of luminal and basal cells, respectively. MCF7 cells, bearing a CD44^neg^/Ep-CAM^pos^/E-cadherin^pos^ phenotype, have been classified as luminal-epithelial and weakly metastatic^19^. Despite of their epithelial origin, MDA-MB-231 cells, presenting a 85 ± 5% of CD44^ + ^/CD24^−^ population, positive to CD105 and negative for both Ep-CAM and E-cadherin staining, are classified as mesenchymal-like phenotype with tendency to metastasize^19^. This cell line over-expresses GLUT1 and typically exhibits Warburg effect characteristics as demonstrated in a xenograft mouse model, by correlating the acidification of the external tumor microenvironment to the lactic acid production^20^. Moreover, this occurrence was proved to be the key driver for local invasion from both primary and metastatic tumor masses, with consequent enhanced growth conditions^21^,^22^.

Combining the knowledge on GLUT1 expression patterns with the Warburg effect, our goal was to investigate on the differences between mesenchymal- and epithelial like cancer cells. Due to their large application in cancer diagnosis and treatment, we used glucose-coated MNPs as vectors introduced in the culture medium. Regarding MNP uptake, we proved a distinctive behavior between epithelial- and mesenchymal-like cells, thus allowing us to discriminate them in co-culture. Interestingly, tuning the glucose concentration in the medium could further enhance this difference.

## Results

### Glucose coated CoFe_2_O_4_ NPs characterization and biocompatibility validation

**Figure 1:**
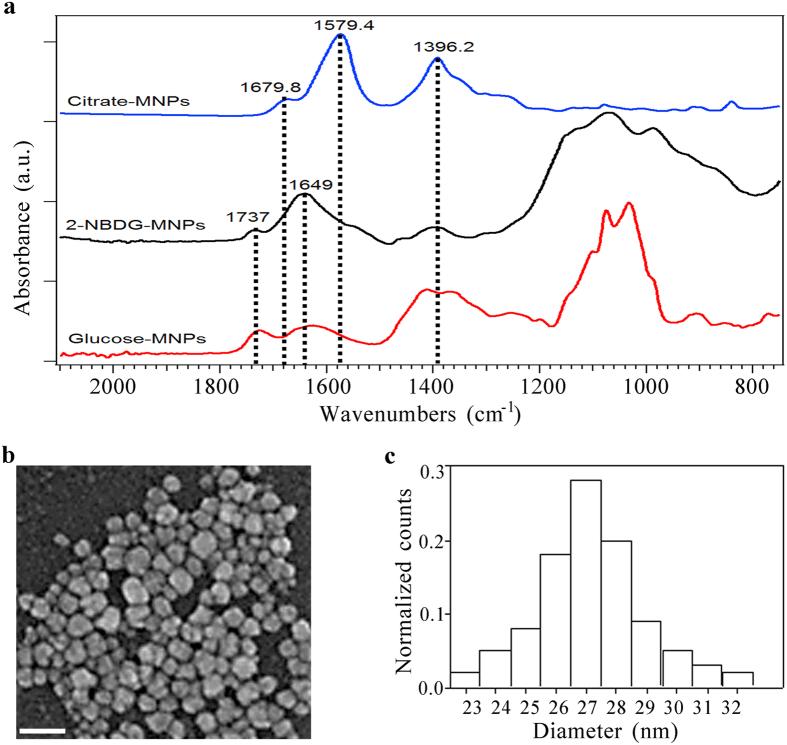
CoFe_2_O_4_ NPs covalently functionalized with glucose and 2-NBDG. (**a**) ATR-FTIR absorbance spectra: citrate coated MNPs (blue), 2-NBDG coated MNPs (black), D-glucose coated MNPs. The covalent reaction between MNPs and the glucose molecules can be appreciated by the peak at 1737 cm^−1^, associated with the ester bonding formation. (**b**) A representative SEM image of CoFe_2_O_4_ NPs; scale bar 80 nm. (**c**) MNPs size distribution (counts normalized to the total number of measured particles). MNPs mean size : 27 ± 3 nm.

To have the control on the MNPs properties and their chemical functionalization we synthesized CoFe_2_O_4_ NPs in the laboratory, following the protocol described in the Methods section. The covalent binding of glucose and its fluorescent analogue, the 2–2-(*N*-(7-Nitrobenz-2-oxa-1,3-diazol-4-yl)Amino)-2-Deoxyglucose (2-NBDG), to the CoFe_2_O_4_ NPs has been chemically characterized using Attenuated Total Reflection–Fourier Transform Infra-Red (ATR-FTIR) spectroscopy. The absorbance spectra of citrate stabilized MNPs (blue line), 2-NBDG functionalized MNPs (black line) and glucose MNPs (red line) are reported in Fig. 1a. The ATR spectrum of the citrate stabilized MNPs is characterized by few broad bands, the most intense of which are centered at 1396 cm^−1^ (symmetric stretching of COO^−^) and 1579 cm^−1^ (asymmetric stretching of COO^−^). This vibrational pattern reveals the presence of free carboxylate moieties, while the shifts of the carbonyl group of carboxylic moieties from 1710 cm^−1^, in free solution, to 1679 cm^−1^ proved the partial single bond character of the C=O group and consequently the chemisorptions of carboxylate ions onto the MNPs surface^23^,^24^. The coordination band was further shifted as a consequence of the functionalization with the glucose derivative while the carboxylate spectral features were greatly suppressed due to the esterification reaction involving the –OH group of 2-NBDG. A further proof of the covalent ester bonding formation was given by the absorbance peak at 1737 cm^−1^ in MNPs – 2NBDG sample as for the glucose one. The absorbance peaks in the spectral region between 1200 and 900 cm^−1^ are correlated to specific vibrational characteristics of glucose molecule.

The size of MNPs has been measured by Scanning Electron Microscopy (SEM), finding a mean diameter of 27 ± 3 nm for all the three samples (citrate, 2-NBDG and glucose coated NPs) (Fig. 1b,c). Neither the functionalization with 2-NBDG nor the one with D-glucose affected the mean diameter of NPs due to the under-nanometer size of functionalizing agents.

Before proceeding with the MNPs administration to breast cancer cell lines, both MDA-MB-231 and MCF7 cell lines have been evaluated for their specific marker expression confirmation (data provided as Supplementary Fig. 1 and Supplementary Methods). To exclude possible side effects, cell lines have been firstly tested for toxicity related to the use of MNPs functionalized with the 2-NBDG. A 3-(4,5-dimethylthiazol-2-yl)-2,5-diphenyltetrazolium bromide (MTT) assay viability assay has been employed to assess the low cytotoxicity of the 2-NBDG-MNPs administration at 2.5 μg/mL, equivalent to 4.6*10^10^ NPs/mL in accordance to literature^25^ (see Supplementary Fig. 4).

### Glucose concentration in culture medium drives the uptake of glucose coated CoFe_2_O_4_ NPs

**Figure 2:**
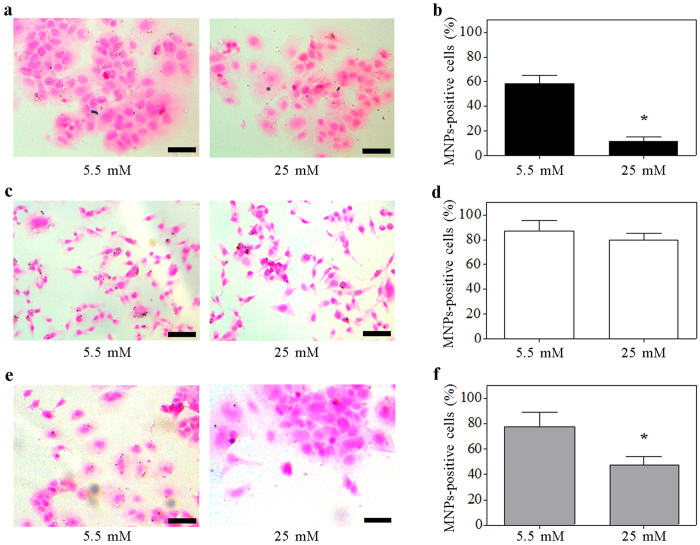
Glucose shell improves the MNPs uptake in breast cancer cell lines. The importance of the MNPs glucose shell has been evaluated at both glucose medium concentrations by counting the Prussian-blue positive cells after the Perls’ iron staining. Representative images of MCF7 (**a**), MDA-MB231 (**c**) and co-culture of MCF7 and MDA-MB231 cells (**e**) in which iron deposits are identified by blue and nuclei are counter-stained by red congo. (**b**,**d,f)** quantification of the fraction of MCF7 (**b**), MDA-MB231 (**d**) and co-culture (**f**) cells showing MNPs internalization (number of cells counted per sample: > 200). Data are presented as mean ± standard deviation. *p < 0.01 vs. 5.5 mM (t-test). Scale bars: 50 μm.

**Figure 3:**
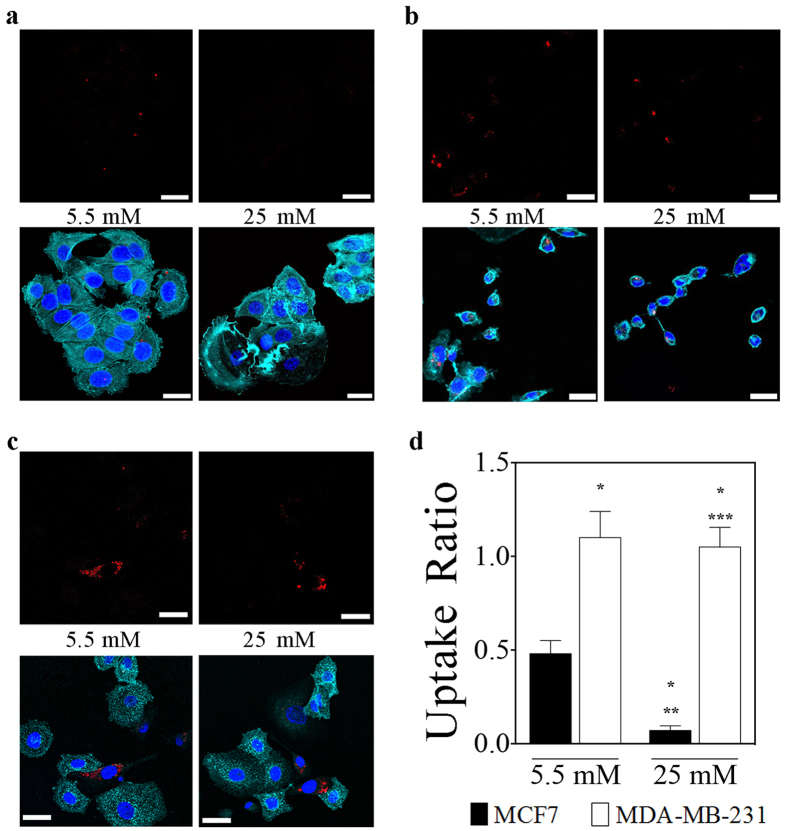
Glucose medium concentration discriminates between mesenchymal-like and epithelial-like breast cancer cells. Data were obtained analyzing by confocal microscope breast cancer cell lines treated with MNPs functionalized with 2-NBDG at the reported glucose concentrations. (**a,b)**, Representative images of MCF7 (**a**) and MDA-MB231 (**b**) treated with 2.5 μg/mL CoFe_2_O_4_–2-NBDG NPs at both glucose medium concentrations. In the upper panels, MNPs are identified by the red fluorescence; in the lower panels, the localization of MNPs (red fluorescence) within the cells is confirmed by the presence of actin filaments (cyan fluorescence of rhodamine-conjugated phalloidin), and nuclei (blue fluorescence of DAPI). MCF7 cells exhibited a low uptake of 2-NBDG functionalized MNPs at 5.5 mM glucose concentration and quasi-null uptake ability at 25 mM glucose concentration. Conversely, MDA-MB-231 cells exhibited a quite constant uptake of 2-NBDG functionalized MNPs at both glucose concentrations. (**c**) representative image of a MCF7/MDA-MB231 co-culture sample; MNPs are evidenced in red while cyan fluorescence identifies the E-cadherin expression (epithelial marker) and nuclei are depicted by the blue fluorescence of DAPI. (**d**) Quantification of the CoFe_2_O_4_–2-NBDG NPs uptake by MCF7 and MDA-MB-231 cells, at both glucose medium concentration. Data are presented as mean ± standard deviation. *, **, ***p < 0.01 vs. the first, the second and the third column, respectively (One way Anova followed by post-hoc Bonferroni test). Scale bars: 50 μm.

**Figure 4:**
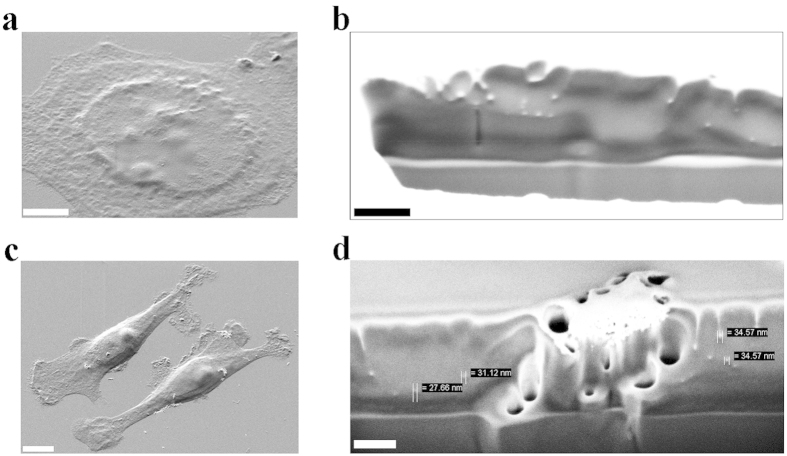
MNPs internalization investigated by the FIB-SEM technique. (**a**) a SEM image of a MCF7 cell before undergoing the precise milling process by the FIB gun. Scale bar: 5 μm. (**b**) a cytoplasm portion of the MCF7 cell depicted in (**a**), after the milling procedure and the zooming in, where several white and rounded objects are visible. In this picture a high brightness/contrast ratio was set to better appreciate the separation line between cytoplasm (grey) and external membrane (white). The white particles in these images have a range diameter of 20–40 nm, in agreement with the MNPs diameter expectations. The bottom part, in grey, is the glass surface where the cells grew. Scale bar: 200 nm. (**c**) a SEM image of two MDA-MB-231 cells before undergo the precise milling process. Scale bar: 5 μm. (**d**) the cell in (**c**), after the FIB-guided milling process, displays a MNPs aggregate and several single particles inside the cytoplasm. The bottom part is, as before, the glass surface. Scale bar: 200 nm.

To assess differences in the glucose uptake between mesenchymal- and epithelial- like tumor cells, we investigated the ability of MDA-MB-231 and MCF7 cells in internalizing glucose-modified MNPs. The shift of glucose concentration from low to high was already proven to promote the proliferation of mammary cells^26^. Here, we analyzed MNPs uptake to prove salient disparities between epithelial-like and mesenchymal-like cells. Three different techniques have been employed: Perls’ iron staining (Fig. 2 and Supplementary Fig. 5), fluorescence confocal microscopy (Fig. 3) and FIB cell milling (Fig. 4). Perl’s iron staining was firstly used to assess the presence of MNPs inside the cells through the recognition of the iron. Confocal microscopy was used to quantify the fluorescent MNPs uptake avoiding underestimation and FIB cell milling was finally employed to further demonstrate the presence of internalized glucose-MNPs both as aggregates and single particles.

At normal glucose concentration, both epithelial-like and mesenchymal-like cell lines were found positive to MNPs uptake, although the MNPs internalization by MDA-MB-231 cells was significantly higher with respect to that of MCF7. This difference further increased at high glucose concentration, since the uptake ability of MCF7 cells resulted to be significantly reduced whereas that one of MDA-MB-231 remained unchanged. In fact, as it is shown in Fig. 2b (black bars), the uptake for MCF7 cells decreased by 79.3% (from 58 to 12%) from normal to high glucose concentration, while it remained unchanged for MDA-MB-231 cells (Fig. 2d, white bars).

Data recorded from co-culture samples are reported in Fig. 2e,f. Although the subtype discernment was not achievable, due to the absence of a cell specific marker, counting the Prussian-blue positive cells provided valuable results. The percentage of positive cells at low glucose concentration (77 ± 12%) was lower than that registered for the MDA-MB-231 cells alone, result explicable considering the contribution of MCF7 cells. Moreover, this effect was confirmed at high glucose concentration where a percentage of 47% positive cells were found. In fact, as expected, the number of positive cells in co-culture, due to the contribution of MCF7 cells, was more evident at high than at low glucose concentration (Fig. 2f).

To establish that MNPs were indeed internalized by cells and not simply adherent to the cell surface, their distribution within the cells (previously treated with CoFe_2_O_4_–2-NBDG NPs) were confirmed by collecting z-stack confocal images of cells labeled by DAPI and phalloidin to identify nuclei and cytoplasmic actin filaments, respectively (see Supplementary Movies 1 and 2).

Figure 3a,b show representative fluorescence images of MCF7 and MDA-MB-231 cells in which internalized fluorescent CoFe_2_O_4_–2-NBDG NPs (red color) are illustrated to exemplify the distinct behavior of the two cell lines. Specifically, at normal glucose concentration both epithelial-like and mesenchymal-like cell lines were found positive to MNPs uptake, with pronounced particle internalization by MDA-MB-231. On the contrary, at high glucose concentration, the uptake ability of MCF7 cells was reduced whereas that of MDA-MB-231 remained almost unchanged (Fig. 3d). MNPs uptake has been tested also in co-cultures of the two breast cancer cell lines using an anti E-cadherin antibody to specifically detect, within the co-culture, MCF7 cells (see Methods). MNPs-fluorescence quantification by confocal microscopy of the E-cadherin –positive and –negative cells revealed comparable uptake values to those observed in single cultures (Fig. 3c and Supplementary Movie 3). Specifically, there was a significant increased uptake of MNPs in the E-Cadherin negative cells with respect to those positive for the epithelial surface markers at both glucose concentrations. Moreover, as for the MCF7 in single culture, the uptake of MNPs in E-cadherin positive cells significantly decreased when cultured at higher glucose concentration (Fig. 3c,d).

Since the resolution of the confocal microscope is unable to resolve single MNPs we decided to quantitatively evaluate the uptake difference between the two cell lines by measuring the total fluorescence emitted by the cells rather than counting the fluorescent particles. Specifically, analyzing samples treated or not by MNPs, we defined the uptake ratio, R_u_, (see methods) as a metrics to evaluate MNPs uptake. The quantitative analysis confirmed that at low glucose concentration, MDA-MB-231 cells showed a significantly higher glucose uptake than MCF7 cells. Nevertheless, increasing the glucose concentration the uptake did not change significantly for MDA-MB-231 cells (from R_u_ = 1.10 to R_u_ = 1.05, *p* = n.s., Fig. 3d) while it decreased by 85.4% in MCF7 cells (from R_u_ = 0.48 to R_u_ = 0.07, *p < 0.01*, Fig. 3d).

The increased uptake of MNPs by MDA-MB-231 cells, with respect to MCF7 cells, could be explained by the different amount of GLUT1 protein expressed by these two breast cancer cell lines (Supplementary Figs 2 and 3). In fact, at 5 mM glucose concentration the relative amount of GLUT1 was about 3.8 times higher in the mesenchymal-like representative cell line respect to the epithelial-like one, while at high glucose concentration (25 mM) this ratio increased to 10 after 48 hours (see Supplementary Figs 2 and 3). Consistently, the glucose modified MNPs uptake paralleled the GLUT1 expression levels. At high glucose concentration, the uptake ability of MCF7 cells was significantly reduced whereas that one of MDA-MB-231 remained unchanged.

The further confirmation of MNPs internalization by breast cancer cell lines was achieved by the FIB precise milling technique coupled with SEM inspection (see Methods). Two representative images of MCF7 and MDA-MB-231 cell lines, treated with CoFe_2_O_4_–2-NBDG NPs and that subsequently underwent to the cytoplasm precise FIB milling, are provided in Fig. 4. In the MCF7 case (Fig. 4a) an image of the inner part of the cytoplasm is reported, where several white objects can be appreciated (Fig. 4b). These white particles showed an average size of 30 nm, in accordance with the measured mean diameter of functionalized CoFe_2_O_4_ NPs. The white color of these is due to the electronic contrast that belongs to the different atomic mass of metals (cobalt and iron in this case), usually completely absent into cells. In support, also not-treated cells underwent the milling process and such evidence was not registered at all. In Fig. 4c an example of MDA-MB-231 cells showing few big MNPs clusters (around 500 nm) on the membrane can be seen. When the cells were investigated by the FIB-SEM technique, beside the aggregate, it was also possible to assess the presence of several internalized single-particles, pointed out by the measuring bars, with a size in accordance with that of MNPs, in Fig. 4d.

### The glucose functionalization is necessary for MNPs uptake

The data obtained on CoFe_2_O_4_–2-NBDG NPs uptake provided the insights for further investigation about the internalization process and its reliance on glucose shell. To exclude that MNPs internalization was a process independent from the glucose functionalization, citrate MNPs have been used as controls. When the fraction of cells internalizing MNPs was evaluated by the Perls’ iron staining, with respect to citrate MNPs, a significantly increased fraction of cells resulted to internalize CoFe_2_O_4_–2-NBDG and CoFe_2_O_4_–D-glucose NPs, with no differences between these two latter groups (see Supplementary Fig. 6).

In order to better appreciate the MNPs uptake process, a time dependent analysis was carried out revealing a saturation value at 2 hours for both breast cancer cell lines. In more detail, the MCF7 samples evidenced an increasing uptake of 2-NBDG and D-glucose coated MNPs at low glucose concentrations with about 60% of positive cells after 120 minutes (Supplementary Information Fig. 6a). The glucose-independent uptake of citrate-coated MNPs by MCF7 cells ranged between 10 and 20% after 2 hours of incubation. Hence, the 2-NBDG (or glucose) vs. citrate coated MNPs intake at 5.5 mM concentration was significantly different in MCF7. Conversely, at high glucose concentration, a quite-similar number of positive cells has been registered regardless of the functionalization, ranging from 10 to 18%, leading to the hypothesis of a completely glucose-independent uptake mechanism at this concentration in MCF-7 cells (Supplementary Information Fig. 6b).

Considering MDA-MB-231 samples, the estimation of total Prussian-blue positive cells confirmed a time-dependent internalization trend at both glucose medium concentrations, with a peak, as for the epithelial-like counterpart, at 2 hours (Supplementary Information Fig. 6c,d). In the case of citrate coated MNPs, the total number of positive cells was ranging between 5 and 20%, depending on the incubation interval. Differently from MCF7 cells, MDA-MB-231 cells maintained a high percentage of 2-NBDG/D-glucose MNPs-positive cells at both low and high glucose concentration, being the internalization always significantly superior to that of the citrate counterpart. Comparing 2-NBDG with D-glucose functionalization, no remarkable differences were appreciated among low and high glucose-concentrations: 87% and 83% at 5.5 mM; 77% and 81% at 2 mM (Supplementary Information Fig. 6c,d).

### Glucose transporter 1 guides the uptake of CoFe_2_O_4_–2-NBDG NPs

To confirm the role played by GLUT1 and explain the differences in the observed high glucose uptake, GLUT1 specific inhibition was carried out using both a chemical compound (STF-31) and a biological inhibition (small interference RNA, siRNA, transfection).

The STF-31 cytotoxicity has been evaluated by MTT assay, executed at different concentrations and time points, before proceeding with the MNPs uptake inhibition studies (see Supplementary Methods and Supplementary Fig. 7).

**Figure 5:**
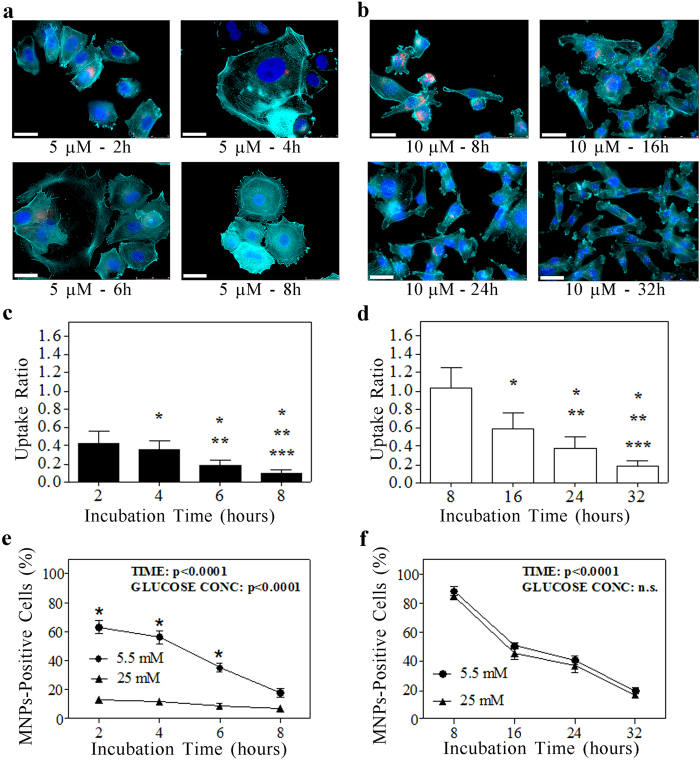
Selective inhibition of GLUT1 transporters suppresses the MNPs glucose shell effect. (**a,b)** representative examples of MCF7 (**a**) and MDA-MB-231 (**b**) fluorescence images after the administration of STF-31 inhibitor at different time points (glucose medium concentration: 5.5 mM). In red are evidenced the MNPs, in cyan the actin filaments and in blue the nuclei. Scale bars: 30 μm. (**c,d)** histograms represent the uptake ratio (mean ± standard deviation) of MCF7 (**c**) and MDA-MB231 (**d**) at different time points. *, **, ***p < 0.01 vs. the first, the second and the third column, respectively (One-way Anova followed by Bonferroni post-hoc test). (**e,f**) Quantification of the MNPs-uptake as fraction of cells stained by the Perls’ iron after GLUT1 inhibition in MCF7 cells (STF-31 administered at 5 μm) (**e**) and MDA-MB-231 cells (STF-31 administered at 10 μm) (**f**). About a 20% of positive cells were found in both cancer subtypes at the latter time points, probably due to unspecific uptake processes. A two-way ANOVA test was used to examine the influence of time and glucose-concentration on MNPs uptake in either MCF7 (**e**) or MDA-MB-231 (**f**). *p < 0.0001 vs. 25 mM.

Drug concentrations of 5 μm for MCF7 and 10 μm for MDA-MB-231 have been chosen as the lowest concentration inducing a time-dependent MNPs uptake reduction without observing a marked cytotoxicity for each cell type. Indeed, at double concentrations (10 μm for the epithelial-like subtype and 20 μm for the mesenchymal-like one) an earlier uptake inhibition was observed together with an increased cytotoxicity. The MNPs uptake inhibition was quantified by fluorescence assay for the CoFe_2_O_4_–2-NBDG NPs and by Perls’ staining for the D-glucose. STF-31-mediated inhibition revealed an essential involvement of the GLUT1 transporters in the glucose modified MNPs internalization in both breast cancer cell lines. In the case of MFC7, the first statistically significant uptake inhibition effect was achieved after 4 hours with the maximum reached after 8 hours (Fig. 5a,c). In contrast, MDA-MB-231 cells revealed a significant CoFe_2_O_4_–2-NBDG NPs uptake diminution after 16 hours of 10 μM STF-31 incubation, reaching the maximum effect at 32 hours (Fig. 5b,d). The fluorescence CoFe_2_O_4_–2-NBDG NPs evaluation at the last incubation time points furnished, for both cell lines, a really low “Uptake Ratio” value: 0.1 for MCF7 and 0.2 for MDA-MB-231 (Fig. 5c,d). In order to confirm these data, Perls’ iron staining was performed as well, finding about 20% of Prussian-blue positive cells after 8 and 32 hours for MCF7 and MDA-MB-231, respectively (see Fig. 5e,f). Again, no differences between the kinetic of inhibition of MNPs internalization was observed for MDA-MB-231-7 at 5.5 and 25 mM.

**Figure 6:**
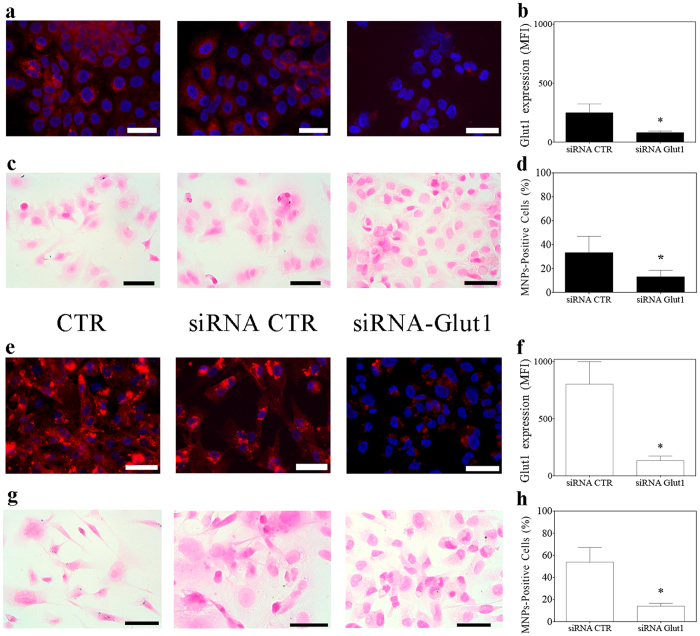
Direct inhibition of GLUT1 transporter via siRNA transfection reduces MNPs uptake in breast cancer cells. The effect of GLUT1 silencing via specific siRNA transfection is reported both as GLUT1 protein diminished expression (**a**,**b**,**e**,**f**) and as 2-NBDG MNPs diminished uptake (**c,d,g,h**). **(a,e)** epi-fluorescence images of GLUT1 protein expression (in red) in MCF7 (**a**) and MDA-MB-231 (**e**) cells, respectively. The decrease of GLUT1 protein expression on breast cancer cells, has been quantified as mean fluorescence intensity (MFI). (**b**,**f**) the fluorescence quantification of images in **a** and **e**, respectively, is reported for both treatment (siRNA CTR: scrambled small interfering RNA). (**c**,**g**) The effect of GLUT1 silencing on 2-NBDG MNPs uptake has been evaluated by counting the fraction of Prussian-blue positive cells after the Perls’ iron staining. (**d**,**h**): quantification of the fraction of MCF7 (**d**), MDA-MB231 (**h**) cells showing MNPs internalization (number of cells counted per sample: > 300). All data are presented as mean ± standard deviation. *p < 0.01 vs. siRNA CTR samples (t-test). Scale bars: 50 μm.

The biological inhibition of GLUT1 protein expression has been obtained by transfecting a specific siRNA against GLUT1 mRNA and the inhibition of MNPs uptake has been evaluated 72 hours after transfection. As shown in Fig. 6, the specific inhibition of GLUT1 protein synthesis determines a consequent lower amount of GLUT1 protein presented on membranes. This diminished protein level, in both breast cancer cell lines, is associated with a significant decrease in the 2-NBDG modified MNPs internalization. Specifically, after GLUT1 siRNA administration, MCF7 and MDA-MB-231 cells showed about 13% and 15% of MNPs positive cells, respectively (Fig. 6d,h), corresponding to a 78% and 81% fold decrease in the corresponding uptake capacity, respectively.

### CoFe_2_O_4_–2-NBDG NPs irradiation permits to obtain hyperthermia effects into mesenchymal-like breast cancer cells

**Figure 7:**
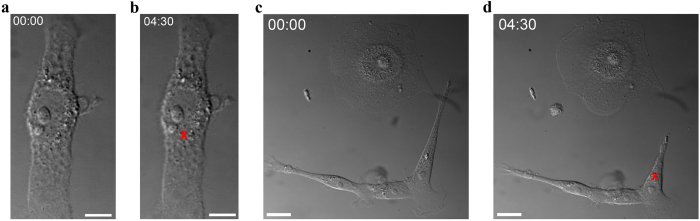
CoFe_2_O_4_–2-NBDG NPs lead mesenchymal-like breast cancer cells to deep modification by hyperthermia. (**a**) a MDA-MB-231 cell in a co-culture environment, where no MNPs have been previously administered. The IR-laser focusing for 4:30 minutes, on the position indicated by the red cross, did not evidence any effect, as it can be seen by comparing the cell before (**a**) and after (**b**) irradiation. Scale bars: 5 μm. For details see Supplementary Movie 8. (**c,d)** few MDA-MB-231 cells and a MCF7 cell in a co-culture environment, after the administration of CoFe_2_O_4_–2-NBDG NPs, before **(c)** and after **(d)** the hyperthermia treatment. In **(d**) a cell shape modification provoked by the localized heating, for 4:30 minutes, can be appreciated. The continuous wave IR-laser beam was focused on MNPs (indicated by the red cross). Scale bars: 20 μm. For details see Supplementary Movie 4.

Specific internalization of CoFe_2_O_4_–2-NBDG NPs has been exploited for hyperthermia studies, taking advantage of the low IR light cell absorption against the high MNPs absorption. The presence of MNPs inside cells was double checked by the light scattered by the MNPs when irradiated with an IR laser beam and by the fluorescence emitted by the MNPs (see Supplementary Movies 4–7). An IR laser beam was focused on a spot of about 4 μm^2^ on the cell area occupied by MNPs. The laser power, on cell target, was increased from 0 to 15 mW by steps of 1 mW. As an effect of localized heating, cells reacted as a function of MNPs number and irradiation time. Hyperthermia targeted cells were recorded in order to observe different heating signals like: bubbles formation, cell shape modification or more drastic effects as cell death. With the purpose to better appreciate the MDA-MB-231 cell shape modification as consequence of the localized heating, the cell contour before and after the hyperthermia was outlined from three movies. These data are provided as Supplementary Fig. 8. To discard the effect of the laser beam on target cells MNPs per se, we focused the laser beam on a sample of MDA-MB-231 cells not treated with MNPs at the maximum power (15 mW) for almost 5 minutes. As illustrated in Fig. 7a,b no damaging effects have been observed (see also Supplementary Movie 8). On the contrary, the MNPs-treated samples exhibited visible effects correlated to the localized heating induced by MNPs the IR light absorption by MNPs during irradiation (Fig. 7c,d; Supplementary Movies 4–7).

## Discussion

In the last decade, several types of NPs have been developed and proposed in the medical research field. Although diverse size and core-shell properties have been suggested and deeply investigated for *in vivo* purposes, several limitations slowed down their clinical applications^27^. In particular, their pronounced tropism for filter organs, with the consequent accumulation and cytotoxicity, enormously delayed the translation into therapy. On the contrary, from a diagnostic point of view, MNPs received very much attention for the development of new and efficient rare cancer-cells enrichment techniques^28^. In this field the only FDA-approved method to detect and count rare circulating tumor cells (CTCs) in metastatic breast, colon and prostate cancer is CellSearch (Veridex)^29^. This method is based on the selection of circulating cells expressing the epithelial specific EpCam antigen. Since this latter is quite low expressed by White Blood Cells (WBCs), it received an extraordinary attention to detect tumor cells of epithelial origin both for diagnostics and in targeted treatment approaches^30^,^31^. However, since the expression of EpCam can be lost by cells in EMT^32^, this method can fail in detecting mesenchymal CTCs. To detect mesenchymal CTC it has been so far proposed a multi-parametric approach based on the combined expression of markers useful for the recognition of mesenchymal cancer cells: CD15^33^, HER-2^34^, CD34^35^, CD44^35^, CD45^36^, CD47^36^, Plastin-3^37^, Vimentin^38^ are the most diffused biomarkers used to enrich and identify CTCs of mesenchymal-origin. These aggressive and metastatic-prone cells own a higher glucose metabolism exhibiting the Warburg effect (as described in the introduction), with a consequently higher demand of energy and glucose uptake needs. The main glucose uptake way is via the glucose transporter channels (Glut family) and their expression is particularly sustained in the mesenchymal cancer subtype.

Here we introduced a MNPs-based method that in principle could provide a simple and low-cost implementation to identify GLUT1-overexpressing cells. Exploiting the glucose cell metabolism, the glucose modified MNPs developed in this work allowed targeting the highly metabolic active breast cancer cells. Epithelial-like and mesenchymal-like cell models have been investigated and compared from a metabolic point of view, leading to the conclusion that at low glucose condition they both internalized glucose-modified MNPs, even though with an appreciable disparity due to their relative GLUT1 protein expression. Increasing the medium glucose level to a non-physiological concentration (25 mM) reflected into an even higher difference between the two cell models tested. Indeed the demonstration of a very well distinguished MNPs uptake ability at 25 mM concentrated glucose could lead in the next future to new feasible solutions in CTCs diagnostics. Moreover metabolism-based methods could implement the current diagnostic techniques by simplifying and improving the mesenchymal-cancer state definition, as demonstrated by GLUT1 immunostaining.

In this work we provided advancements to the current knowledge by the development of a new metabolic assessment of cancer cells, based on the use of MNPs. In particular, we presented a successful functionalization of CoFe_2_O_4_ NPs with glucose molecules and its fluorescent analogue, the 2-NBDG, where the covalent linkage was confirmed by FTIR spectroscopy and fluorescence optical microscopy. The cobalt ferrite nanoparticles due to their higher magnetization characteristics, with respect to normal iron oxide nanoparticles^39^, and the low cytotoxicity exhibited may allow to decrease the concentrations and hence becoming suitable also for future *in vivo* applications. The added value of glucose shell could strongly boost the cancer-targeted therapy allowing to selectively penetrating the tumor and hence reducing the accumulation in non-pathological tissues.

The glucose functionalization of MNPs allowed us to target the more metabolic active breast cancer cell line, *in vitro*, discerning it from the less metabolic active one. Indeed, the GLUT1 overexpressing cell line (MDA-MB-231) internalized a statistically significant higher amount of glucose-coated MNPs, with respect to the MCF7 counterpart, at both glucose medium concentrations assessed. Actually, the most remarkable result was obtained at 25 mM concentration, since at this concentration, with respect to 5.5 mM glucose, the epithelial-like cells lost three/fourth of their ability to uptake glucose coated MNPs as a consequence of the reduced GLUT1 protein expression. Considering the GLUT1 binding affinity for glucose similar in the two cancer cell lines, results can be interpreted as a consequence of a different modulation of GLUT1 expression in the two cell lines as a consequence of changes in the glucose concentration. Even after GLUT1 inhibition, both with chemical and biological methods, a variable percentage in the range of 15 to 20% of cells still continued to internalize glucose-modified-MNPs. This behavior could be interpreted as to be associated with a non-GLUT1 dependent mechanism, introducing the possibility that also other Glut-family members could be important for the MNPs uptake mechanism. For instance GLUT3, GLUT14 and GLUT12 could be involved by observing their relative gene expression (see GEO dataset, GEO accession number: GSE41445) even if GLUT3 is known to be mainly involved in neurons and there are just few evidences about GLUT12 involvement in mammary tumors^40^.

By the use of SEM the MNPs uptake has been confirmed, at nanometer resolution, for both cell types. Focused Ion Beam (FIB) milling, as reported in Fig. 4, made precise investigation of cell cytoplasm and nucleus possible. The recurring occurrence observed, with each technique and confirmed by SEM analysis, was the presence of bigger MNPs aggregates on MDA-MB-231 cells and smaller ones on MCF7 at 5.5 mM glucose concentration. Considering this latter aspect, it could be hypothesized that at a glucose concentration of 25 mM, there is a low interaction of the membrane proteins (in particular glucose transporters) with the MNPs shell due to the glucose-demand saturation. The low interaction strength exerted by MCF7 cells towards MNPs confirms also the already described lower amount of GLUT1 protein expression, with respect to mesenchymal-like counterpart^41^.

Although our evidences strongly suggest that the internalization mechanism is Glut-protein dependent, it is however still unclear how MNPs cross the membrane. In some preliminary experiments we observed, by an immuno-staining study, a slight co-localization between MNPs and lysosome markers. Indeed endocytosis might be a more feasible way for metal nanoparticles to be internalized after interacting with glucose transporters. Nevertheless, further experiments are required to adequately address this hypothesis.

The GLUT1 selective inhibition obtained by STF-31 and gene silencing definitely helped in correlating the MNPs-specific uptake to the activity of this glucose transporter channel in both the tested cancer cell lines. However, the inhibition of MNPs uptake in the mesenchymal-like subtype required, with respect to that in MCF7 cells, an increased STF-31 concentration (about twice) and incubation time (about 4 times), while by siRNA no concentration difference were required. Anyway, at the used concentration, STF-31 revealed a low cytotoxicity in both cell lines until 32 hours of incubation, leading to a marked cytotoxicity only after 72 hours of treatment (Supplementary Information Fig. 7).

In addition to their possible use to detect mesenchymal-like tumor cells, the peculiar tropism of glucose-coated MNPs for mesenchymal-like cells, suggest their employment in therapeutic applications such as in targeted hyperthermia treatments^42^. Based on this hypothesis, here we presented an IR-laser-guided way for single-cell localized overheating. MNPs absorbance in IR allowed transferring these effects to the cell cytoplasm and consequently observing the cell reaction. Cell shape contractions, contour modifications, intra-cytoplasmic bubbles formation, cytoplasm rearrangements and cell death were the main observed occurrences (Supplementary Fig. 8 and Supplementary Movies 4–7 for details). Control cells (not previously treated by MNPs), subjected to IR-laser beam irradiation both on cytoplasm and nucleus, did not show any damaging evidence during and after this procedure (Supplementary Movie 8).

A classic approach in the field of thermotherapy is the use of oscillating magnetic field with precise frequency (100 kHz–1 MHz) and intensity (10–50 mT). Effectively the combination of the low administered MNPs concentration and the related heating-caused effects, coupled with cancer live imaging techniques could convey a significant improvement in cancer field. In this work via the exploitation of glucose coated MNPs, we selectively induced a localized heating on more metabolic active cells, the mesenchymal-like breast cancer cell line MDA-MB-231, providing interesting insights for future applications, especially because selectively targeting the population of cells considered more difficult to remove by conventional chemotherapy strategy because of an intrinsically enhanced drug-resistance. By consent, metal-based NPs are nowadays investigated for targeted heating therapy by the irradiation of precise areas with X-rays^43^. Due to the substantial metal-NPs to cell absorption ratio, in those wavelengths, several applications are now in development in order to face this challenge. The CoFe_2_O_4_–2-NBDG NPs developed in this work are perfectly in accordance with this front thanks to the low cytotoxicity exerted and the high tropism for the metabolic active cancer cells.

With the purpose to develop an implementation method for cancer diagnostic applications, here we also tested the glucose coated MNPs on WBCs obtained from healthy donors. WBC samples were incubated for 2 hours in the presence of CoFe_2_O_4_–2-NBDG NPs in order to observe their uptake ability. Remarkably, WBCs did not show any significant MNPs internalization (Supplementary Fig. 9), supporting the possible application of glucose-coated MNPs in *in vitro* assays aimed at specifically recognizing GLUT1-overexpressing CTCs. This possibility could further improve the current CTCs detection methods, since it focuses the attention on the mesenchymal population, considered to be characterized by cancer stem cell properties and thus truly responsible of tumor maintenance, recurrence and metastasis^44^.

In conclusion, we think we have developed MNPs able to specifically recognize tumor cell subpopulations taking advantage of their distinctive deregulated metabolic pathways (Warburg effect), confirming the link between a more aggressive mesenchymal-like phenotype and a higher demand of energy. This will open the way to develop inexpensive *in vitro* assays and *in vivo* therapeutic approaches aimed at specifically recognizing/targeting the population of tumor cells considered to be endowed with the highest metastatic and drug-resistance potential.

## Methods

### Synthesis and Characterization of CoFe_2_O_4_ NPs

CoFe_2_O_4_ NPs were synthesized accordingly to Massart method^45^. The protocol was partially modified, as we previous described^46^, in order to have citrate-coated MNPs with improved stability in physiological solution, which are more suitable for cellular environment. The citrate coordination of MNPs was used for covalent functionalization of MNPs with glucose molecule and its fluorescent analogue, the 2-NBDG (Life Technologies). The esterification reaction between the free carboxylic group of citrate and the more reactive hydroxyl group on 2-NBDG molecule has been achieved using HCl as catalyzing agent. The normal D-glucose molecule was also used as control sample to confirm the successful functionalization of the MNPs. The esterification was carried out by mixing 0.5 mg/mL of citrate-coated CoFe_2_O_4_ NPs with 1:300 wt/wt aqueous solution of 14 mM 2-NBDG (26.6 mM for D-glucose) in the presence of HCl (3 mM). The solution was kept at room temperature under stirring for at least 2 hours, and then NPs were pelleted employing a permanent magnet, washed 3 times with a buffer solution (10 mM HEPES, 107 mM NaCl, 5.3 mM NaOH, pH 7.4) and stored in bi-distilled water at 4 °C.

ATR–FTIR spectroscopy has been chosen to evaluate the CoFe_2_O_4_ NPs covalent functionalization. Measurements were carried out at the SISSI Beamline of the Elettra Synchrotron (Trieste, Italy) employing a Vertex 70 (Bruker® Co.) purged with nitrogen and a DTGS (Deuterated Tri Glycine Sulfate) detector. MIRacle Single Reflection ATR box (PIKE Technologies) equipped with a diamond IRE (Internal Reflection Element) was used for experimental purposes. A 5 μL drop of aqueous sample was placed onto the crystal and the measurements were repeated until the combination band of bending and vibrational modes of liquid water (centered at 0 ~ 2150 cm^−1^) disappeared. The background was collected on the clean IRE. Spectra were acquired averaging 128 scans with a spectral resolution of 4 cm^−1^.

In order to assess the mean diameter distribution of the MNPs a SEM (Supra 450, Zeiss, Germany) analysis has been performed. MNPs samples were prepared by drying under nitrogen flux a drop of 1 μM concentrated CoFe_2_O_4_ NPs on silica wafer. Samples were then washed twice with drops of milliQ water (electric resistance: 18 mΩ) and dried again by nitrogen blow. The SEM images were analyzed with the “Analyze Particles” tool of ImageJ^47^ for the NPs size evaluation after setting up their brightness/contrast parameters.

### Cell lines culture

MDA-MB-231 and MCF7 breast cancer cell lines were purchased from ATCC (ATCC numbers HTB-26 and HTB-22 respectively) and stored in liquid nitrogen. The phenotype of cultured cells has been evaluated by both flow-cytometry and immunofluorescence. The cells have been cultured in Dulbecco’s Modified Eagle Medium with 5.5 mM glucose (Low Glucose DMEM, Life Technologies) supplemented by a 10% Fetal Bovine Serum in a fully humidified atmosphere of 5% CO_2_ at 37 °C. To evaluate MNPs uptake and GLUT1 expression, MCF-7 and MDA-MB-231 were cultured either in DMEM LG (Low-glucose, 5.5 mM) medium with 10% fetal bovine serum or in DMEM HG (High-glucose DMEM 25 mM, Life Technologies) medium with 10% fetal bovine serum; cell culture medium was changed every 24 hours. The treatment in both standard and hyperglycemic conditions has been evaluated at 24 (T1) and 48 hours (T2).

### Immunofluorescence analysis

For immunofluorescence analyses, MDA-MB-231 and MCF7 cells were fixed in 4% buffered paraformaldehyde and permeabilized with 0.1% Triton X-100. Expression of vimentin (clone V9, Dako), cytokeratins 8–18–19 (clone 5D3, BioGenex), and the estrogen receptor alpha (clone SP1, ACZON) was evaluated by indirect immunofluorescence staining, using Alexa A555-labeled secondary antibodies. The Glucose Transporter GLUT1 was detected using an anti-GLUT-1 rabbit monoclonal antibody labeled with Alexa 647 dye (clone EPR3915, Abcam). DAPI was used to detect nuclei. All images were collected using a Leica DMI6000 B (Leica Microsystems, Germany) utilizing a 40X/63X oil immersion objective (numerical aperture: 1.25 and 1.40 respectively), keeping constant the acquisition parameters. GLUT1 immunostaining was quantified by the ImageJ software. For each specified condition, all time points were evaluated in triplicate, calculating the mean fluorescence intensity of at least 300 cells.

### Flow-cytometry analysis

MCF7 and MDA-MB-231 were detached by Tryple-Express and incubated with the properly conjugated antibodies, following vendor instructions. Properly conjugated isotype-matched antibodies were used as a negative control. The analysis was performed by FACSCanto (BD Bioscience), See Supplementary Materials for details.

### CoFe_2_O_4_ NPs Administration on Breast Cancer Cell Lines

MNPs treatments were carried out on cells growth in either low or high glucose DMEM. After an incubation period of 24 hours, at 37 °C in a humidified 5% CO_2_ incubator. Cancer cells were treated for 2 hours with 2.5 μg/mL of water dispersed CoFe_2_O_4_ solution. Finally, the cells were fixed in a 4% Paraformaldehyde (PFA) solution for 15 minutes at room temperature. Fixed MCF7, MDA-MB-231 and co-culture samples were washed with PBS and membranes permeabilized by 10 minutes incubation with 0.1% TritonX-100 solution. Single cultures of MDA-MB-231 and MCF7 cells were labeled by 1:400 phalloidin (Rhodamine conjugated, λex = 555 nm, Life Technologies) to highlight actin filaments, and by DAPI to identify nuclei. On the contrary, co-culture samples were stained overnight at 4 °C with anti-E-cadherin antibody (1:100 rabbit, Dako) to specifically recognize epithelial cell membranes^48^. After incubation, samples were stained with a secondary anti-rabbit antibody, Cy-7 conjugated (donkey, Abcam). Nuclei were labeled by DAPI.

### Microscopy Evaluation of CoFe_2_O_4_ NPs Uptake

The fluorescence of CoFe_2_O_4_–2-NBDG NPs uptake by MDA-MB-231 and MCF7 cells have been assessed by a confocal laser microscope (DMRE, Leica, Germany) equipped with a 63X oil immersion objective (numerical aperture: 1.40) or a 40X oil immersion objective (numerical aperture: 1.25). In order to cover the entire cell height, a range of about 3–4 μm, for each sample, was captured by a z-stacking interval of 250 nm. The confocal microscopy technique has been chosen in order to associate the presence of MNPs (as fluorescence emitted by the 2-NBDG functionalizing agent) at the same focus plan of the actin filaments, allowing to confirm the MNPs internalization by the cells. The specific 2-NBDG excitation wavelength is in the blue region (λ = 480 nm), while the emission is in the green one (λ = 510–550 nm)^49^. However, in the Figures MNPs are shown in the pseudocolor red to better appreciate their presence. The analysis of the total fluorescence content of each cell was performed by ImageJ “ROI Manager” after single-cell contour selection by the “freehand selection” tool, for each sample. The quantification was performed on the image reporting the average value of fluorescence in the 2-NBDG channel, relative to the total confocal images recorded. The fluorescence coefficient, *C*_*t,n*_, has been calculated for treated (*t*) and non-treated (*n*) samples respectively:
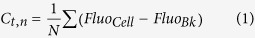
where, FluoCell represents the fluorescence intensity of a single selected cell for the channel related to the CoFe_2_O_4_–2-NBDG NPs emission, FluoBk is the background intensity corresponding to the same cell-contour area and N is the total number of analyzed cells (N = 200 cells from almost 20 regions, per sample). The uptake ratio, *R*_*u*_, is then calculated as:
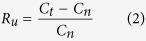


To evaluate the internalization of non-fluorescent MNPs, the Perls’ iron staining, that produces a Prussian-blue deposition in presence of reduced iron, was chosen^50^. The Perls’ iron staining has been carried out by a semi-automated instrument (Artysan™ Link Pro, Dako), on PFA-fixed samples either permeabilized or not by a membrane permeabilization procedure (10 minutes with a 0.1% TritonX-100 solution), in order to assess intracellular -or cell surface bound- MNPs, respectively (see Supplementary Fig. 5).

To assess the glucose function in MNPs uptake and so avoid possible 2-NBDG dye contribution, D-glucose and citrate coated CoFe_2_O_4_ NPs were used. The uptake process, for each MNPs functionalization type, has been tracked after 5, 15, 30, 60, 120, 180 and 240 minutes, in both glucose medium conditions. The samples were prepared on duplicate and the experiments were repeated three times. The quantification of cells positive for internalized MNPs has been performed on pictures acquired via optical microscope (Leica DMD 108 equipped with a 10×, 20× and 40× objectives, numerical aperture 0.40, 0.70 and 0.95, respectively). Cells were counted as positive in case of Prussian blue deposition (compatible as to be MNPs) regardless of the number of events per cell. The quantifications are reported as total counted Prussian-blue-cells respect to the total amount of cells in the area. At least 200 cells per sample were analyzed. ImageJ software was employed to overlay single-channel images and adjust the brightness/contrast settings, while to compose the final images Adobe Photoshop was utilized.

### Uptake of MNPs investigated by SEM-FIB technique

Breast cancer cell lines have been further analyzed at the nanometer level by SEM, to evaluate the presence on intracellular MNPs. Cell samples were fixed with 4% PFA for 15 minutes and dehydrated by increasing concentration of Et-OH (from 30% to 99.9%, purchased from Sigma-Aldrich Co LLC). Samples were dried by gently nitrogen blow and coated with a 20 nm thick Chromium film by sputtering. Samples were transferred in a Cross Beam Microscope (Zeiss International, Germany) for Gallium Focused Ion Beam (FIB) precise milling and Scanning Electron Microscopy (SEM) inspection. The chromium coating, via sputtering, allowed delineating the cell morphology and afterward the FIB cuts were performed on random positions, onto cells, and on visible aggregates as well, in order to examine whether and where the MNPs were internalized.

### Pharmacological inhibition of the Glucose Transporter Channel 1

To inhibit GLUT1, breast cancer cell lines have been treated with the specific inhibitor STF-31 (Sigma-Aldrich Co. LLC). The evaluation of toxicity has been performed via the MTT assay, whereas the effect of STF-31 on NPs uptake was evaluated both by Perls’ iron staining and immuno-fluorescence microscopy as described above. The protocol followed for the STF-31 toxicity evaluation is described in Supplementary Methods. The uptake inhibition effect has been observed at 5 μM and 10 μM concentrated inhibitor in MCF7 and MDA-MB-231, respectively. The time points analyzed were 2, 4, 6 and 8 hours for MCF7 and 8, 16, 24 and 32 hours for MDA-MB-231 and MNPs were added 2 hours before the PFA fixation for each time step. Each treatment was carried in 2 wells (in a 24 well-plate) and repeated 3 times.

### Suppression of GLUT1 expression by siRNA delivery

A validated siRNA against GLUT1 gene (SLC2A1, *Invitrogen*) was transfected into MDA-MB-231 and MCF-7 cells at a concentration of 10 nM. The cells were then transfected with the transfection reagent Lipofectamine 2000 (*Invitrogen*) in accordance with the manufacturer’s instructions. The transfection reagent and the GLUT1 siRNA were incubated with the cells in Opti-MEM®(reduced serum media, *Gibco*) for 72 hours. Control cells were incubated in the same volume of transfection reagent with a control siRNA (*Invitrogen*). The samples were prepared on triplicate and the experiments were repeated four times. Cellular levels of GLUT1 were quantified by immunofluorescence staining and FACS analysis (details are described in the previous paragraphs).

### Cell Local Heating Through NPs Laser Beam Absorption

A continuous-wave infrared (IR) laser beam (IPG Photonics) was focused through the microscope lens (Nikon, 60X water immersion objective, numerical aperture: 1.25) onto the selected MNPs-bearing cell. The setup was built on a Nikon microscope (Eclipse TE2000-E, Nikon, Japan) and images recorded on CMOS camera (Hamamatsu ORCA-Flash 4.0). The wavelength of the laser was 1064 nm, for which cell adsorption is minimum. In each recorded movie an IR filter was inserted in order to better appreciate the effects caused by the laser. It was, moreover, used to avoid the IR-laser entrance into recording device and thus allowing good video quality. The power on the sample has been regulated from 0 to 15 mW to evaluate the increasing outcomes. Cobalt ferrite nanoparticles absorbance in IR is lower than visible or UV wavelength^51^ but in the same time much higher than that of the cells^52^.

### Statistics

Data were tested for normal distribution using the Kolmogorov-Smirnov test and therefore characteristics of the population have been described using means ± standard deviation. Paired T-test or unpaired test, as appropriate, was used to compare continuous variables between two groups. One-way Anova followed by Bonferroni post-test were used to compare more than two groups. Two-way Anova followed by Bonferroni post-test was employed to evaluate the effects of both incubation time and glucose concentration on STF31-treated breast cancer cell lines. P values less than 0.05 were considered significant (Prism, version 4.0c).

## Additional Information

**How to cite this article**: Venturelli, L. *et al*. Glucose is a key driver for Glut1-mediated nanoparticles internalization in breast cancer cells. *Sci. Rep.* **6**, 21629; doi: 10.1038/srep21629 (2016).

## Supplementary Material

Supplementary Information

Supplementary Movie 1

Supplementary Movie 2

Supplementary Movie 3

Supplementary Movie 4

Supplementary Movie 5

Supplementary Movie 6

Supplementary Movie 7

Supplementary Movie 8
