# Supplementary material for: Glucose is a key driver for GLUT1-mediated nanoparticles internalization in breast cancer cells
Source: Sci Rep. 2016 Feb 22;6:21629. doi: 10.1038/srep21629 (PMC4761954; doi:10.1038/srep21629)
Supplement: Supplementary Information [file srep21629-s1.doc]

**Glucose is a key driver for Glut1-mediated nanoparticles internalization in breast cancer cells**

Leonardo Venturelli^1,2,3^, Silvia Nappini^2^, Michela Bulfoni^3^, Giuseppe Gianfranceschi^3^, Simone Dal Zilio^2^, Giovanna Coceano^1,2^, Fabio Del Ben^1,3^, Matteo Turetta^3^, Giacinto Scoles^3^, Lisa Vaccari^4^, Daniela Cesselli^3^ and Dan Cojoc^2^.

Corresponding authors: [daniela.cesselli@uniud.it](mailto:daniela.cesselli@uniud.it); cojoc@iom.cnr.it.

^1^ PhD School of Nanotechnology, Department of Physics, Via Valerio 2, I-34127, University of Trieste, Trieste, Italy.

^2^ CNR – Institute of Materials, Area Science Park-Basovizza, S.S. 14, Km 163.5, I-34149 Trieste, Italy.

^3^ Department of Medical and Biological Sciences, Piazzale Kolbe 2, I-33100, University of Udine, Udine, Italy.

^4^ Elettra Synchrotron Trieste, SISSI beamline, Area Science Park-Basovizza, , S.S. 14, Km 163.5, I-34149 Trieste, Italy.

**SUPPLEMENTARY INFORMATION**

**
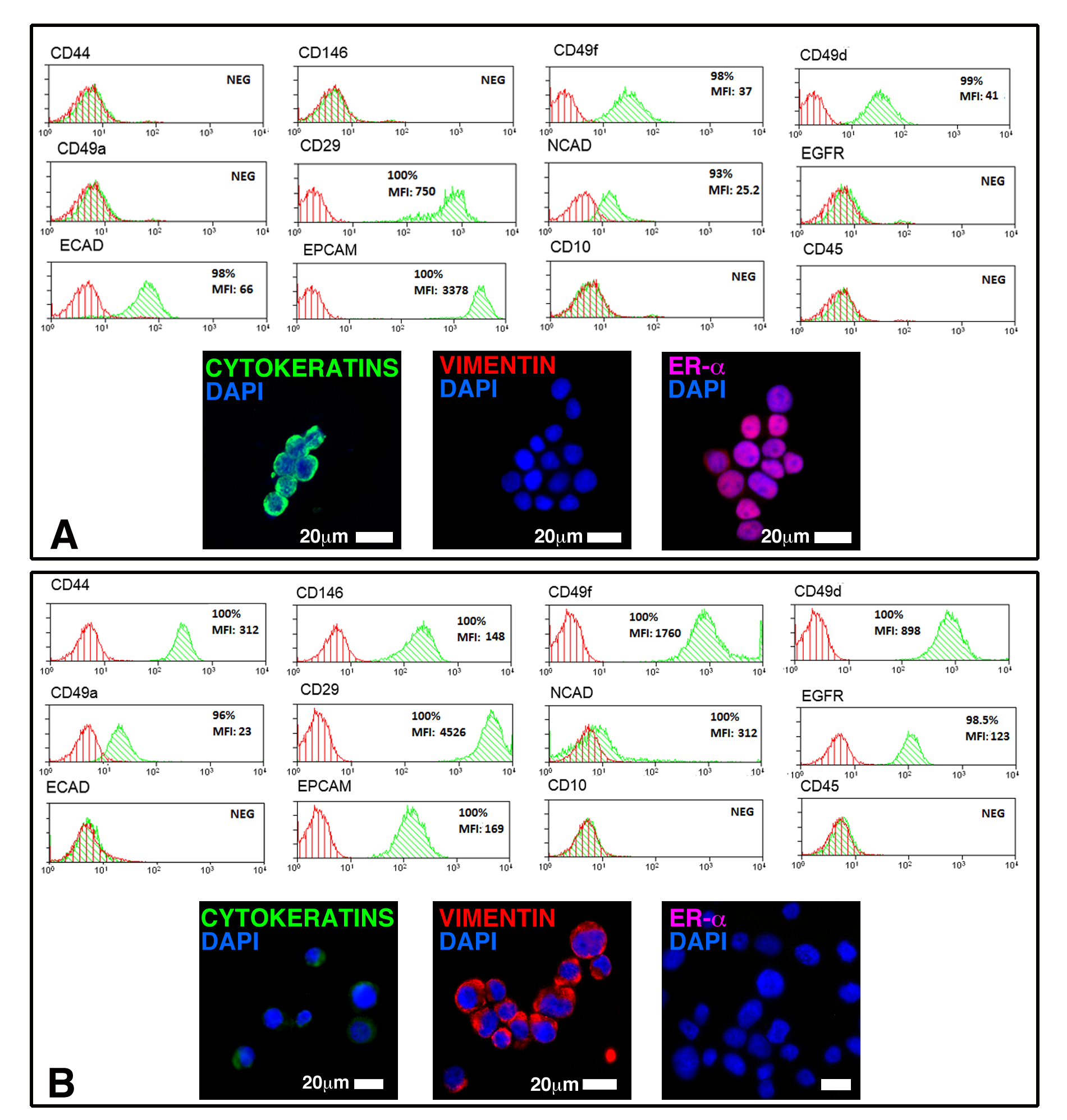
**

**Supplementary Figure 1** **| Breast cancer cell lines: phenotype assessment.** The phenotype of MCF-7 **(A)** and MDA-MB-231 **(B)** was evaluated both by flow-cytometry (upper panels) and immunofluorescence (lower panels). In the FACS histograms, the percentage of positive cells and the mean intensity fluorescence (MFI) for each marker tesed are indicated in the corresponding upper right corner. Green lines indicate the positive staining cells, while the red ones indicate the isotype-matched antibody control. ERα = estrogen receptor alpha.


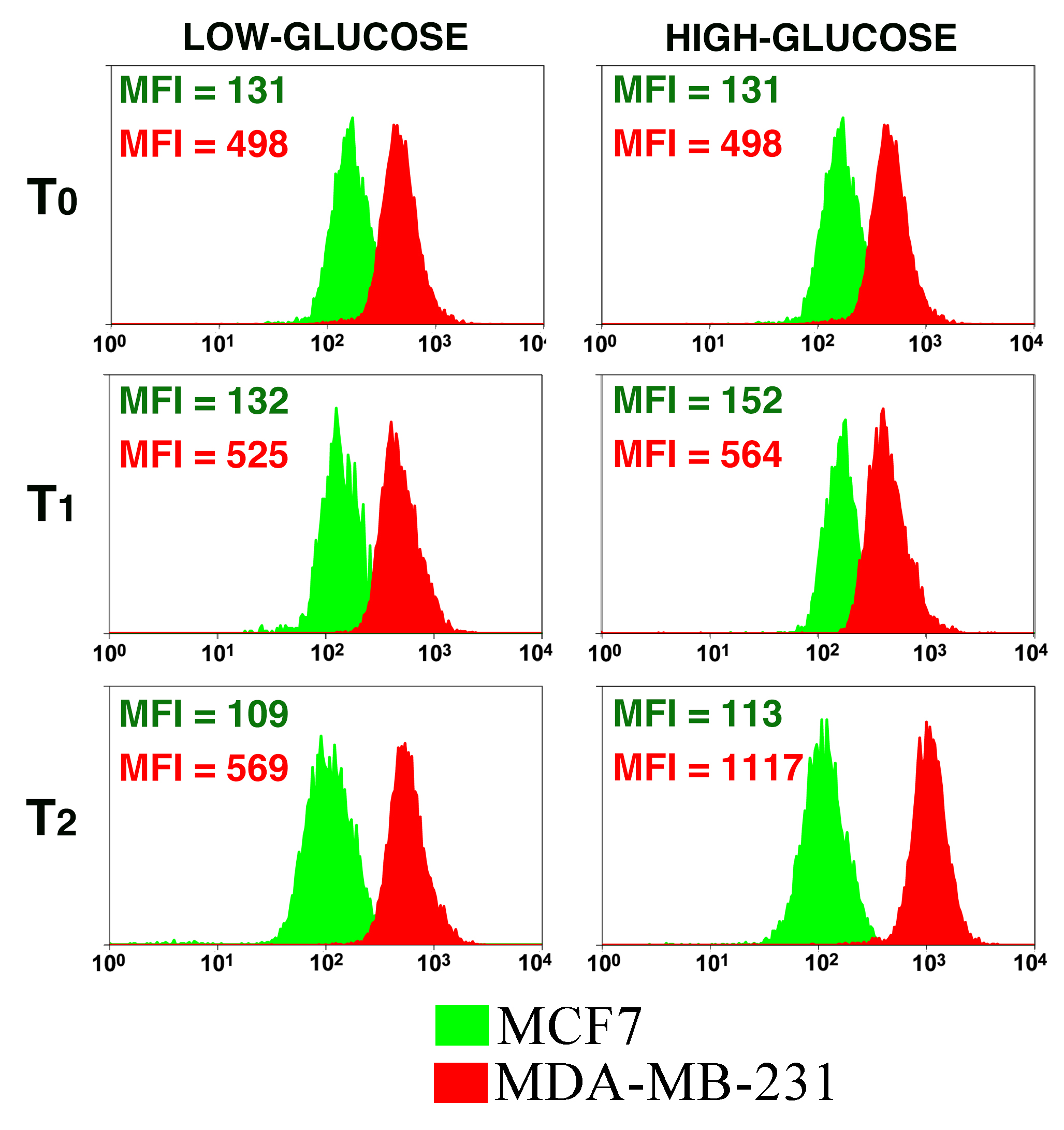


**Supplementary Figure 2 | Glut 1 expression in breast cancer cell lines analyzed by FACS.** The Glut1 expression level of the two breast cancer cell lines has been firstly evaluated by flow-cytometry. The Glut1 expression level ratio of MDA-MB-231 respect to MCF7 goes from 3.8 times at T0 to 5.2 at T2 in low glucose DMEM and from 3.8 to 10 times at T2 in high glucose DMEM. T0 is the starting point, T1 is 24 and T2 is 48 hours after seeding, respectively.


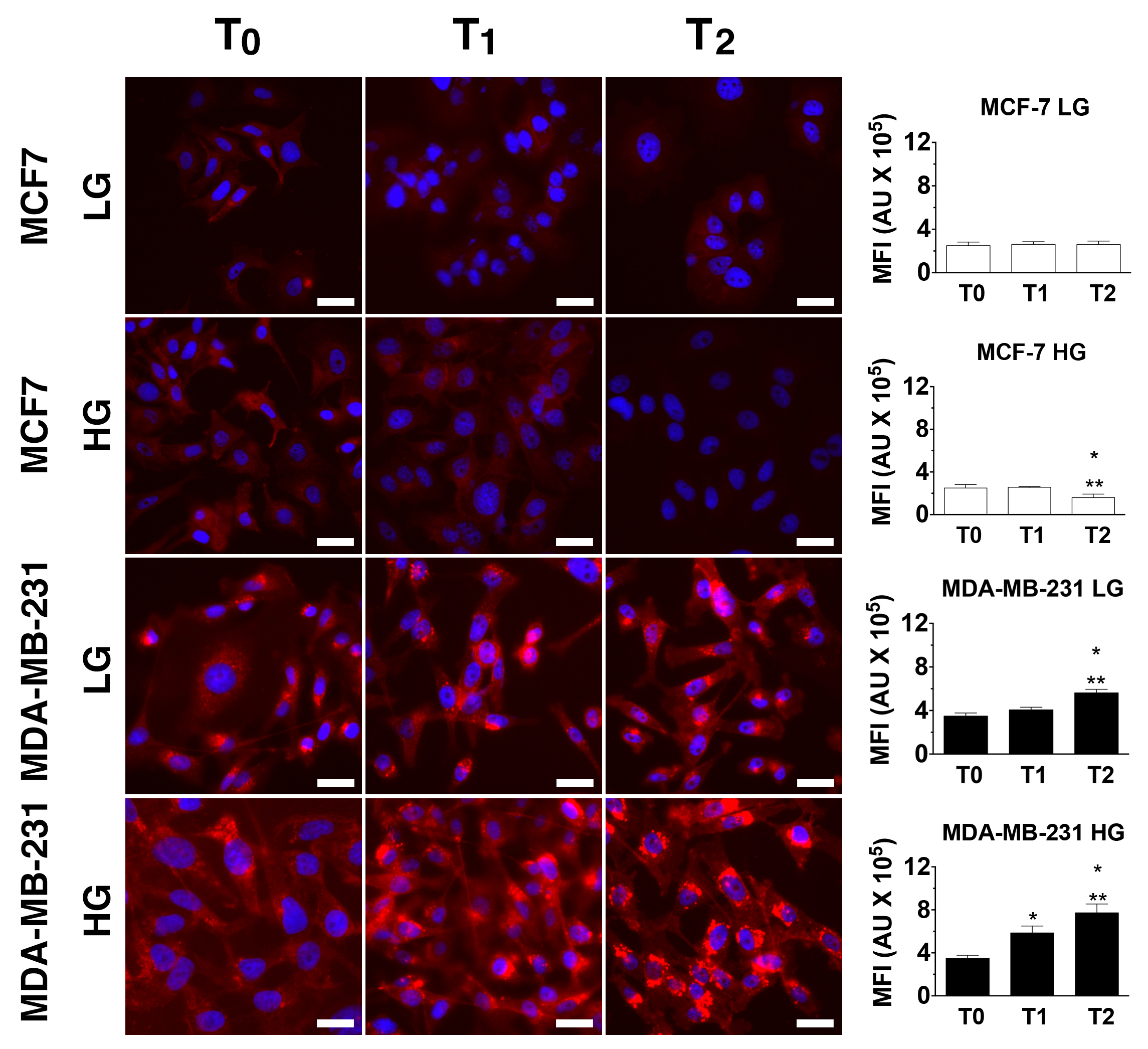


**Supplementary Figure 3 | Glucose-medium concentration tunes Glut1 expression in breast cancer cells**. We tested the effect of the glucose concentration in the medium on the modulation of Glut1 protein expression (red fluorescence). Incubation in Low glucose (LG) and High Glucose (HG) DMEM for 0 (T0), 24 (T1) and 48 (T2) hours causes different Glut1 expression in the cell line tested. The most interesting result is the statistical significant increased level of Glut1 protein in MDA-MB-231 both at 24 (T1) and 48 (T2) hours. Conversely, MCF7 cells showed a significant decrease in Glut1 expression at 48 hours. Nuclei are depicted by the blue fluorescence of DAPI. *, **, p<0.05 vs. T0 and T1, respectively. Scale bar 30μm.


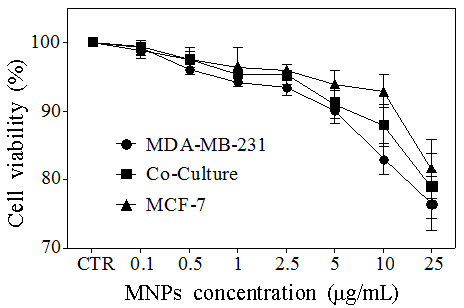


**Supplementary Figure 4 | MNPs toxicity evaluation on breast cancer cell lines**. Cell viability assay for breast cancer cell lines cytotoxicity evaluation after 72 hours of CoFe_2_O_4_ – 2-NBDG NPs administration is reported. The treatments on breast cancer cell lines were carried out in low glucose DMEM (glucose concentration = 5.5 mM)


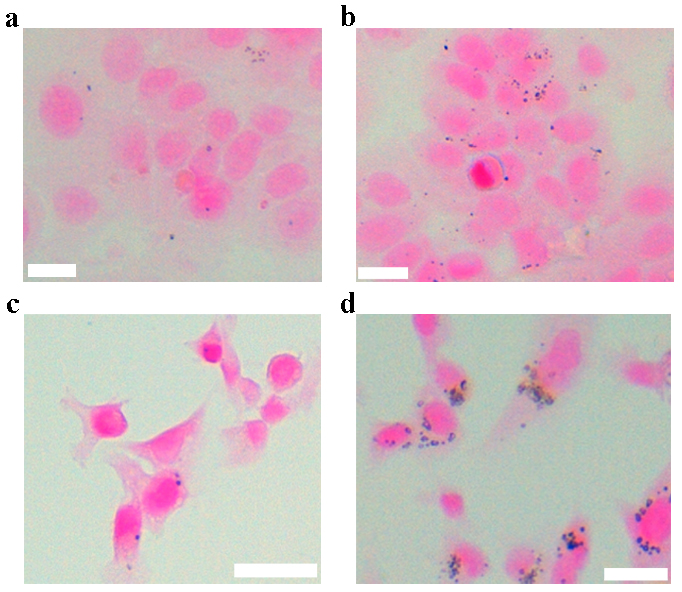


**Supplementary Figure 5 | TritonX-100 effect on Perls’ iron staining**. The fundamental effect in Prussian-blue positive cells enumerating due to the permeabilization step introduced at the beginning of the Perls’ iron staining procedure. **a**: MCF7 cells sample after the Perls’ iron staining without any permeabilization step at the beginning. Only few blue dots on cells can be visible. Scale bars: 30 μm. **b**: MCF7 cell sample after the Perls’ added with the permeabilization step: several Prussian-blue spots can be encountered. **c**: MDA-MB-231 cell sample fixed and colored by hematoxylin/eosin after Perls’ iron staining without the permeabilization step. **d**: MDA-MB-231 cells permeabilized before undergo the Perls’ iron staining: several Prussian-blue spots can be appreciated in the cells cytoplasm. Scale bars: 20 μm.

**
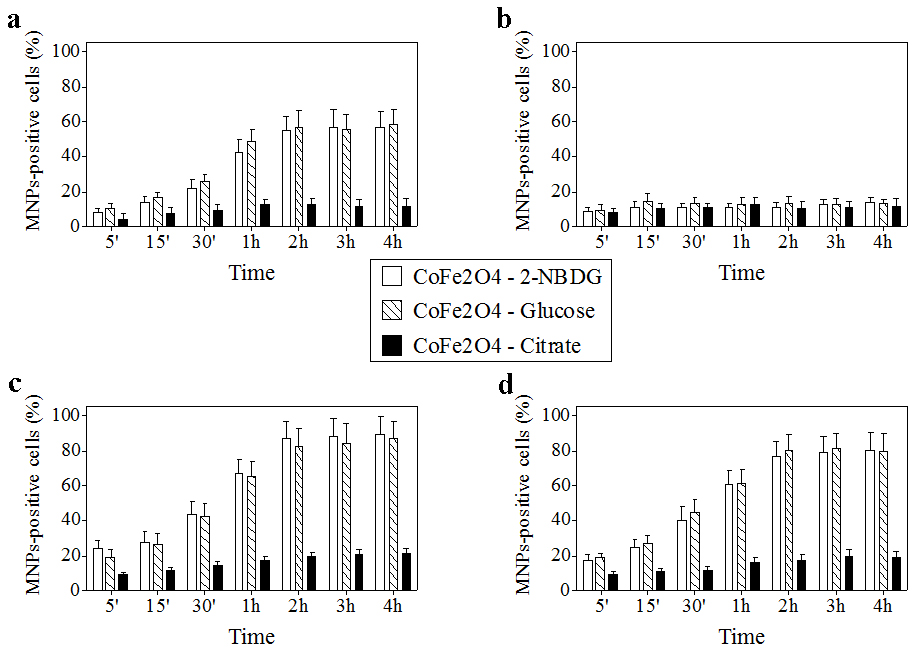
**

**Supplementary Figure 6 | Time-dependent MNPs uptake by breast cancer cell lines.** The quantification of MNPs-positive cells were calculated by Prussian-blue positive cells counting, as described in the main text. The uptake amount of MNPs in each sample was quantified also 3 and 4 hours after the incubation, but any statistically significant improvement was registered respect to the 2 hours incubation. **a**: MCF7 cells treated at 5.5 mM concentrated glucose, where a sort of threshold was reached after 2 hours of incubation with a maximum of less than 60% of positive cells was registered. **b**: MCF7 cells treated at 25 mM where the cells exhibited only MNPs unspecific uptake regardless of the functionalization. **c**: MDA-MB-231 cells at 5.5 mM concentrated glucose inside the culture medium. The number of MNPs-positive cells in the 2-NBDG sample is almost 85% and it is comparable with the D-glucose-MNPs sample after 2 hours of incubation. **d**: MDA-MB-231 cells at 25 mM concentrated glucose inside the culture medium. The MNPs-positive cells in the 2-NBDG sample are comparable to the D-glucose-MNPs and are just under 80%, after 2 hours of incubation. The legend is reported in the middle.

***
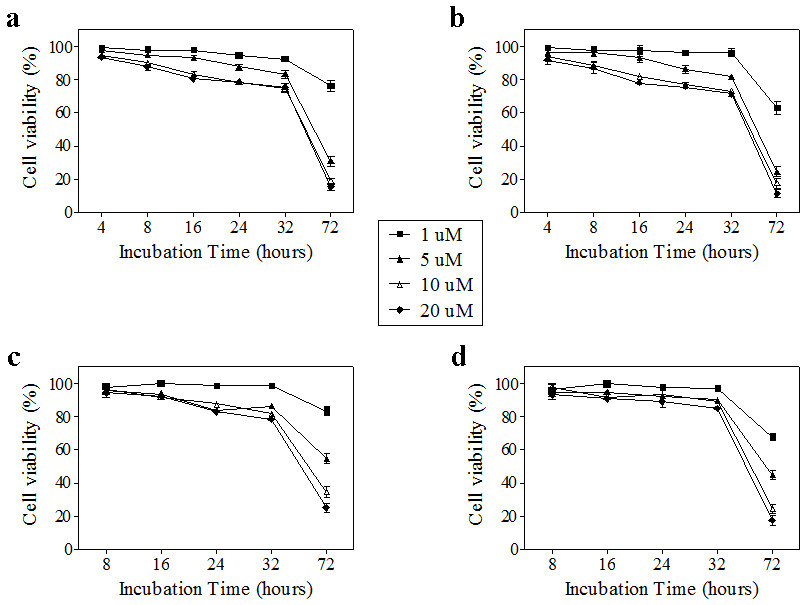
***

**Supplementary Figure 7 | STF-31 cytotoxicity on breast cancer cell lines.** The STF-31 compound tested for the cytotoxicity evaluation on breast cancer cell lines at both glucose medium concentrations via MTT test. **a**: MCF7 cell cultured at 5.5 mM concentrated glucose treated with STF-31 at different concentrations. The Glut1 inhibitor disclosed, after 72 hours, a marked cytotoxicity on MCF7 cells at 5,10 and 20 μM, while a 1 μM just around a 20% of cells was dead. **b**: MCF7 cell cultured at 25 mM concentrated glucose treated with STF-31 at different concentrations. At high glucose concentration the epithelial-like cells showed higher cytotoxicity at 1 μM (40%), while at higher drug concentrations the results were comparable with those obtained in **a**. **c**: MDA-MB-231cell cultured at 5.5 mM concentrated glucose treated with STF-31 at different concentrations. **d**: MDA-MB-231 cell cultured at 25 mM concentrated glucose treated with STF-31 at different concentrations. The mesenchymal-like cells showed a lower sensitivity to the Glut1 inhibition respect to the epithelial-like counterpart, maybe due to their higher expression of Glut1 proteins. The legend is reported in the middle.

**
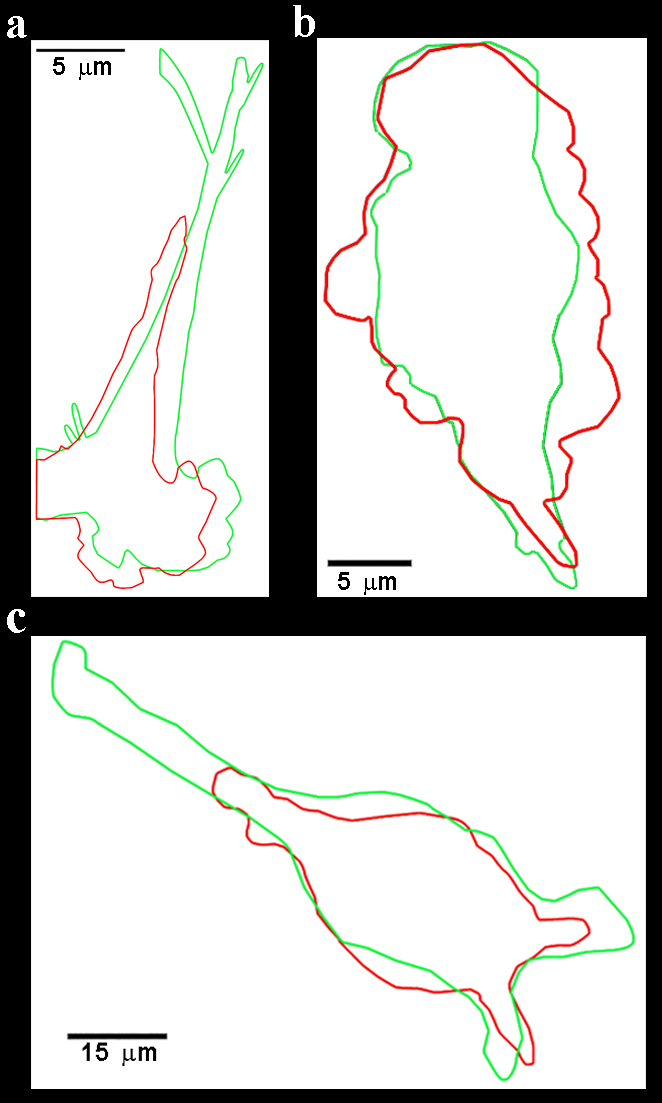
**

**Supplementary Figure 8 | Mesenchymal-like cell contours changing due to the localized overheating.** In green and in red are reported the cells contours regarding the MDA-MB-231 cell shapes before and after the IR-laser localized hyperthermia, respectively. Image in **a** regards the cell recorded in the movie titled “Supplementary Movie 4”. Image in **b** regards the cell recorded in the movie titled “Supplementary Movie 5”. Image in **c** regards the cell recorded in the movie titled “Supplementary Movie 6”. Each scale bar is reported.


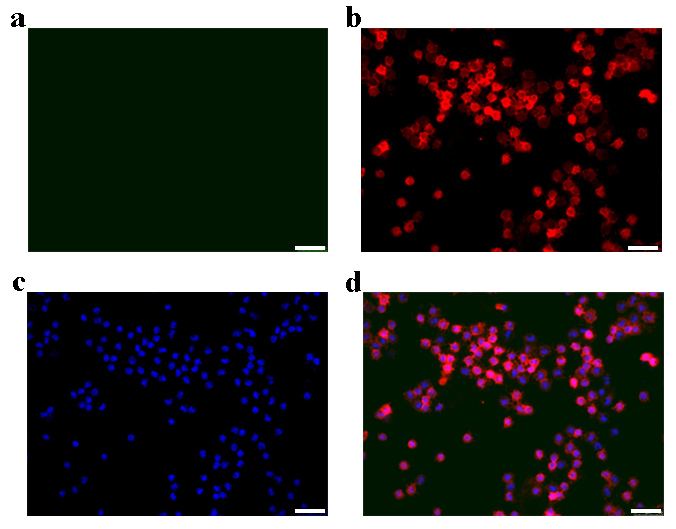


**Supplementary Figure 9 | Healthy WBCs treated with 2.5 μg/mL CoFe_2_O_4_ – 2-NBDG NPs.** **a:** MNPs channel is reported in green, where no MNPs-positive cells were registered. **b**: anti-CD45 Antibody staining for the confirmation of the CD45-positive population. **c**: nuclei (DAPI). **d**: merged images of the three image in a, b and c. Scale bars: 25 μm.

***Supplementary Methods***

**Breast cancer cell lines characterization.** MDA-MB 231 and MCF-7 breast cancer cell lines were tested by flow cytometry to determine their phenotypic markers falling within two different categories: epithelial-like and mesenchymal-like. Cells were analyzed using monoclonal antibodies labeled with phycoerythrin (PE), fluorescein isothiocyanate (FITC), Alexa 647 or allophycocyanine (APC). Antibodies were directed against the following antigens: CD90 (5E10 E-bioscience), CD44 (IM7, Bilegend), CD146 (BD Biosciences), CD49a (TS2/7, Biolegend), CD49d (9F10 Biolegend), CD49f (G0H3, Biolegend), CD10 (BD Biosciences), CD29 (BD Biosciences), CD45 (BD Biosciences), EGFR (AY13, Biolegend), EpCAM (9C4, Biolegend), N-cadherin (8C11, BD Biosciences), E-cadherin (674A, Biolegend), and GLUT-1 (clone EPR3915, Abcam). The respective isotype controls were used for each antibody to verify the binding specificity. Cells were analyzed employing a FACSCantoII (BD Biosciences) flow-cytometer.

**MNPs uptake by healthy white blood cells.** White Blood Cells (WBCs) population was obtained by using the Fraction Collector Omnicoll®, from healthy donor whole blood samples. 1 mL of whole blood was diluted in an equal volume of 1X PBS and then gently added in a FACS tube containing 2mL of Omnicoll. Tube was centrifuged at 500 rcf for 20 minutes. WBCs population was collected and put on gently rotation in a 1.5 mL tubes ferris wheel with 2.5 μg/mL of CoFe_2_O_4_ – 2-NBDG NPs for 2 hours in DMEM low glucose (Life Technologies) at 37°C with a 5% CO_2_ in humidified atmosphere. Then an anti-CD45-PE-Texas Red antibody was added. CD45 is a well known transmembrane protein expressed by leukocytes. After 15 minutes of antibody staining, cells were gently washed and centrifuged at 300 rcf for 3 minutes and the supernatant removed. Pellet was resuspended in PBS 1X solution and a drop of each of them was placed on a cover slip glass slide. The fluorescence signal of the CoFe_2_O_4_ – 2-NBDG NPs was completely absent due to the non-significant amount of MNPs introduced by the WBCs. Representative example images are provided in Supplementary Figure 7.

**Cell viability Assay.** MDA-MB-231 and MCF7 breast cancer cell lines, detached during the exponential phase, were seeded 5·10^3^ per well in a 96-wells plate in low glucose DMEM (Life Technologies). Cells were left to attach for 24 hours and subsequently treated with CoFe_2_O_4_ – 2-NBDG NPs at different concentrations: 0.1, 0.5, 1, 2.5, 5, 10 and 25 μg/mL. Control cells were treated with an equal volume of PBS. Each treatment was carried out through 6 wells and repeated 3 times. Cell viability was analyzed after 72 hours by 3-(4,5-dimethylthiazol-2-yl)-2,5-diphenyltetrazolium bromide (MTT) assay (Life Technologies). Specifically 5 mg/mL solution of MTT was added, 1:10, at the culture medium 3 hours before reading the absorbance at 570nm with a Tecan Infinite 200 PRO (Tecan Trading AG, Switzerland).

**Glut1 inhibition via STF-31 administration.** The Glut1 inhibitor (STF-31 drug) has been tested for cytotoxicity evaluation, before proceeding with the MNPs uptake inhibition studies. The STF-31 has been diluted in DMSO^2^ at the following concentrations: 1, 2, 5, 10, 20 μM. The treatments have been carried out at the following incubation interval: 2, 4, 8, 16, 24, 32 and 72 hours. The cells were seeded 5·10^3^ per well in a 96-well plate in low or high glucose DMEM, depending by the sample, and left to attach for 24 hours before drug administration. The medium was removed and fresh serum-free medium added, with the STF-31 diluted in it. The serum was supplemented at 10% after 4 hours. In the case of 2 and 4 hours intervals, the cells have not received any serum implementation. The cell viability evaluation has been achieved by MTT assay as described in the previous paragraph.

***Supplementary Movies Description***

**Supplementary Movie 1**. Confocal images reconstruction of different z-stacking level of MCF7 sample treated with 2.5 μg/mL of CoFe_2_O_4_ – 2-NBDG NPs at 5.5 mM concentrated glucose. MNPs are in red, actin filaments in cyan and nuclei in blue.

**Supplementary Movie 2**. Confocal images reconstruction of different z-stacking level of MDA-MB-231 sample treated with 2.5 μg/mL of CoFe_2_O_4_ – 2-NBDG NPs at 5.5 mM concentrated glucose. MNPs are in red, actin filaments in cyan and nuclei in blue.

**Supplementary Movie 3**. Confocal images reconstruction of different z-stacking level of breast cancer cell co-culture sample treated with 2.5 μg/mL of CoFe_2_O_4_ – 2-NBDG NPs at 25 mM concentrated glucose. MNPs are in red, E-cadherin in cyan and nuclei in blue.

**Supplementary Movie 4**. It is a movie regarding the hyperthermia effect on MDA-MB-231 cell (previously treated with 2.5 μg/mL of CoFe_2_O_4_ – 2-NBDG NPs) irradiated by a continuous-wave IR laser beam (λ=1064 nm), where a retraction of cellular body is distinctly visible, due to heating of MNPs aggregates, previously internalized by the cell. It can be appreciated both the light scattering from MNPs and the fluorescence light emitted by MNPs.

**Supplementary Movie 5**. The movie regards the hyperthermia effect on MDA-MB-231 cell (previously treated with 2.5 μg/mL of CoFe_2_O_4_ – 2-NBDG NPs) irradiated by a continuous-wave IR laser beam (λ=1064 nm) directly on MNPs aggregates, internalized by the mesenchymal cell at low glucose medium concentration. Hyperthermia effects pertain bubbles formation into whole cells and cytoplasmic rearrangements during the recorded time-interval.

**Supplementary Movie 6**. The movie regards the hyperthermia effect on MDA-MB-231 cell (previously treated with 2.5 μg/mL of CoFe_2_O_4_ – 2-NBDG NPs) irradiated by a continuous-wave IR laser beam (λ=1064 nm) directly on MNPs aggregates, internalized by the mesenchymal cell at 5.5 mM glucose medium. During the movie can be appreciated the cell-prolongation retraction and the complete detachment from the surface. Hyperthermia effects led the cell to the necrosis after few minutes.

**Supplementary Movie 7**. The movie regards the hyperthermia effect on MDA-MB-231 cell (previously treated with 2.5 μg/mL of CoFe_2_O_4_ – 2-NBDG NPs) irradiated by a continuous-wave IR laser beam (λ=1064 nm) directly on MNPs aggregates, internalized by the mesenchymal cell at 25 mM glucose concentrated medium. Hyperthermia effects pertain bubbles formation near MNPs position due to warm caused by IR absorbance of cobalt ferrite NPs. During the movie can be appreciated the scattered light from MNPs (due to laser beam focusing) and the fluorescent emission of 2-NBDG, coating MNPs.

**Supplementary Movie 8**. The movie regards a MDA-MB-231 cell, not treated with CoFe_2_O_4_ – 2-NBDG NPs, irradiated by the same IR laser beam of the previous movies, provided for supporting the concept that localized hyperthermia effects were exclusively attributable to MNPs absorbance and not to laser beam overheating.

***Supplementary References***

1. Boon, K. in *Breast Cancer - Focusing Tumor Microenvironment, Stem cells and Metastasis* (ed. Gunduz, M.) (InTech, 2011). at <http://www.intechopen.com/books/breast-cancer-focusing-tumor-microenvironment-stem-cells-and-metastasis/the-mesenchymal-like-phenotype-of-the-mda-mb-231-cell-line>

2. Chan, D. A. *et al.* Targeting GLUT1 and the Warburg Effect in Renal Cell Carcinoma by Chemical Synthetic Lethality. *Sci. Transl. Med.* **3,** 94ra70–94ra70 (2011).
